# Biópsia Endomiocárdica por Meio de Técnica de Biótomo Rígido e Risco de Regurgitação Tricúspide após Transplante Cardíaco

**DOI:** 10.36660/abc.20240223

**Published:** 2024-10-17

**Authors:** Luís Beck-da-Silva, Leonardo Hennig Bridi, Bruno S. Matte, Felipe Homem Valle

**Affiliations:** 1 Hospital de Clínicas de Porto Alegre Porto Alegre RS Brasil Serviço de Cardiologia - Hospital de Clínicas de Porto Alegre, Porto Alegre, RS – Brasil; 2 Universidade Federal do Rio Grande do Sul Departamento de Medicina Interna Porto Alegre RS Brasil Departamento de Medicina Interna da Universidade Federal do Rio Grande do Sul (UFRGS), Porto Alegre, RS – Brasil

**Keywords:** Biópsia Endomiocárdica, Biótomo, Rígido

## Abstract

A biópsia endomiocárdica (BE) é o procedimento de preferência para o diagnóstico de rejeição pós-transplante cardíaco. A técnica de biótomo rígido tem sido usada devido à sua maior simplicidade e tem sido criticada pelo risco potencial de regurgitação tricúspide (RT). Nosso objetivo foi revisar todas as BEs realizadas por meio dessa técnica em um centro terciário e estimar a taxa de complicações e/ou agravamento por RT.

Estudo transversal, retrospectivo, anterógrado. Os dados foram coletados de 729 BEs realizadas em 55 pacientes pós-transplante cardíaco, com um biótomo rígido Scholten Novatome™ entre setembro de 2012 e março de 2022. Todas as BEs foram realizadas pela veia jugular direita sob anestesia local e por meio de micropunção e orientação por ultrassom. Um total de 729 procedimentos tiveram ecocardiografia realizada antes e depois dos procedimentos. A estimativa da RT foi categorizada como ausente, mínima, leve, moderada e grave. O teste qui-quadrado de McNemar foi usado para analisar o grau de RT pré e pós-BE.

Houve piora suficiente para se tornar RT moderada ou grave pós-biópsia em 2 (0,27%) procedimentos, e houve uma ligeira alteração na RT de mínima para leve em 25 (3,42%) procedimentos. Em 729 BEs percutâneas realizadas com um biótomo rígido, não houve perfuração miocárdica, tamponamento cardíaco ou pneumotórax. Uma morte ocorreu dentro de 24 horas após o procedimento, por motivo desconhecido.

A BE com biótomo rígido é segura e não foi associada à piora da RT no acompanhamento de 729 BEs realizadas após transplante cardíaco.

A taxa geral de complicações, incluindo RT moderada a grave, foi de 0,81%. A taxa de mortalidade foi de 0,14%.

## Introdução

A biópsia endomiocárdica (BE) é o método padrão para detectar rejeição em pacientes submetidos a transplante cardíaco desde a década de 1970.^1^ A técnica de biópsia evoluiu ao longo dos anos seguindo o desenvolvimento e a evolução do transplante cardíaco. A técnica rígida foi desenvolvida pela primeira vez em meados da década de 1970 como um método de BE desenvolvido por Caves e associados,^2^ na Universidade de Stanford, para avaliação da rejeição de aloenxerto cardíaco. O novo biótomo Scholten Novatome™ combina a experiência duradoura reunida desde seu desenvolvimento na década de 70 e a exclusividade de um dispositivo descartável fabricado com material biocompatível. O biótomo tipo Scholten é simples, rápido e seguro de usar. No entanto, ele requer que a válvula tricúspide seja cruzada com a pinça do biótomo para cada coleta de amostra. Para superar essa potencial limitação, uma técnica flexível foi desenvolvida, usando um biótomo flexível que passa por uma longa bainha introdutora, permitindo que a válvula tricúspide seja cruzada apenas uma vez, de modo que todas as amostras sejam coletadas através dessa bainha. No entanto, a literatura carece de uma comparação de ambas as técnicas em relação ao risco de regurgitação tricúspide (RT).

O presente estudo tem como objetivo revisar todas as BEs realizadas pela técnica do biótomo tipo Scholten em um centro terciário e estimar a taxa de complicações e/ou agravamento de RT.

## Método

Trata-se de um estudo transversal, retrospectivo, anterógrado.

**Figura 1:**
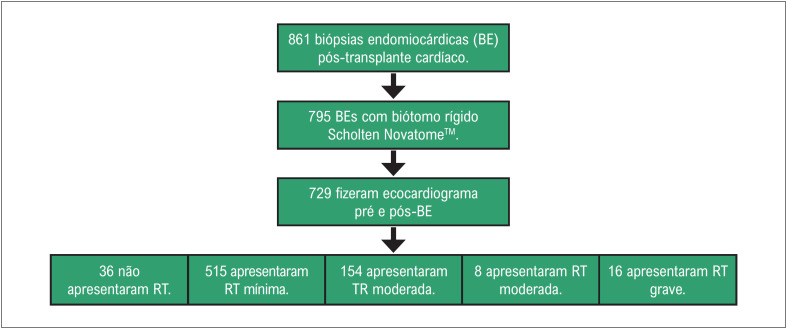
Fluxograma de biópsias endomiocárdicas pós-transplante cardíaco realizadas de setembro de 2012 a março de 2022 incluídas na análise e o grau de regurgitação tricúspide pré-biópsia. RT: regurgitação tricúspide.

Os pacientes foram selecionados por meio de consulta ao sistema eletrônico do hospital (AGHUse). O período de busca das biópsias foi de setembro de 2012 a março de 2022. O filtro utilizado foi o procedimento realizado: BE. A partir da lista contendo todas as biópsias realizadas, o prontuário do paciente foi aberto para verificar o motivo da realização das biópsias miocárdicas. Apenas pacientes pós-transplante cardíaco foram incluídos. Os dados foram coletados de 861 BEs. Destes 861 procedimentos, 795 BEs foram realizadas com biótomo rígido Scholten Novatome™, em 55 pacientes pós-transplante cardíaco. Nossos pacientes pós-transplante cardíaco seguem um protocolo para realização de biópsias semanais no primeiro mês, biópsias quinzenais até o terceiro mês, biópsias mensais até seis meses e a cada dois meses durante o primeiro ano pós-transplante. Procedimentos extras são incluídos em caso de rejeição, conforme necessário. Os dados estudados consistem em 729 BEs realizadas com um biótomo rígido Scholten Novatome™, com ecocardiografia realizada antes e depois das BEs (Figura 1). A estimativa de RT foi avaliada por ecocardiografia e categorizada como ausente e mínima, leve, moderada e grave. Todos os exames ecocardiográficos foram realizados por cardiologistas experientes.

**Figura 2:**
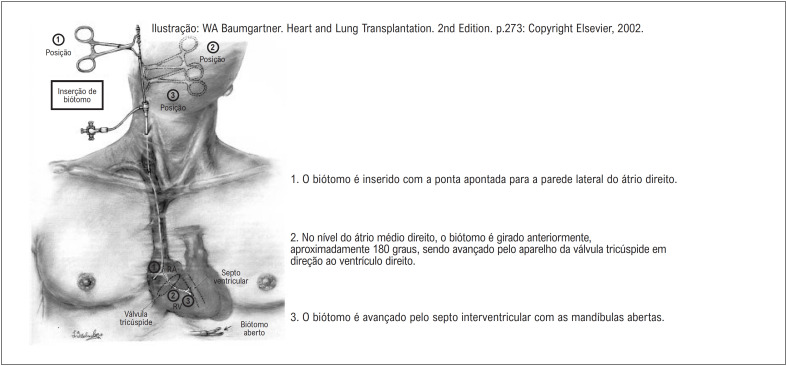
Posicionamento do biótomo rígido para biópsia endomiocárdica.

Todas as 729 BEs foram realizadas pela veia jugular direita sob anestesia local, por meio da técnica de micropunção e sob orientação de ultrassom vascular (Tabela 1). Sob controle fluoroscópico, o biótomo ventricular direito, um cateter de 50 cm de comprimento com diâmetro externo de 2,3 mm, foi avançado para o terço inferior do átrio direito, com suas mandíbulas cortantes voltadas para a direita. A parte portátil do biótomo é um hemostático modificado que abre e fecha as mandíbulas por meio de um fio de acionamento rígido, que também dá ao biótomo sua direcionalidade.^3^ O cateter é girado por sua alça de modo que a ponta fique voltada para o orifício da válvula tricúspide e, então, é lentamente avançado pelo ventrículo direito; uma vez que a válvula tricúspide tenha sido gentilmente cruzada, o biótomo é girado mais medialmente. Quando o septo ventricular direito foi contatado, a resistência e a contração ventricular são sentidas pelo operador. As mandíbulas são então fechadas e o biótomo é retirado firmemente (Figura 2). Uma ou poucas contrações ventriculares prematuras são comuns quando o dispositivo toca o tecido endomiocárdico e no momento da coleta da amostra. A estimativa da RT foi avaliada por ecocardiografia e categorizada como ausente e mínima, leve, moderada e grave.

**Tabela 1:** Características basais

		Todos
Idade (anos) (n=55)		50 (40; 59)
Sexo feminino (n = 55)		19 (35%)
Número de procedimentos por paciente (n=55)		13 (8; 15)
Número de fragmentos por procedimento (n=729)		4 (3; 4)
RT pré-procedimento (n=729)		
	Ausente ou mínima	551 (76%)
	Leve	154 (21%)
	Moderada/grave	24 (3%)

RT: regurgitação tricúspide.

Os dados são apresentados como média ± desvio padrão, mediana (percentil 25; percentil 75) ou n (%).

Um teste qui-quadrado de McNemar foi usado para comparar a RT pré e pós-biópsia. Este teste considera a comparação de alterações pré e pós-procedimentos. Por se tratar de um teste bicaudal, o valor real deste p reflete quaisquer alterações, independentemente de ser em direção à melhora ou piora da RT (Tabela 2).

**Tabela 2:** Distribuição de 729 biópsias endomiocárdicas em relação à regurgitação tricúspide pré e pós-procedimento

		RT pós-biópsia					
		Falta	Mínimo	Leve	Moderado	Grave	Total
RT pré-biópsia	Falta	4	29	2	1	0	36
	Mínimo	4	486	25	0	0	515
	Leve	7	79	68	0	0	154
	Moderado	0	0	0	7	1	8
	Grave	0	14	2	0	0	16
	Total	15	608	97	8	1	729

RT: regurgitação tricúspide.

## Resultados

A idade média dos pacientes foi de 50 anos (Intervalo interquartil 41, 60).

**Figura 3:**
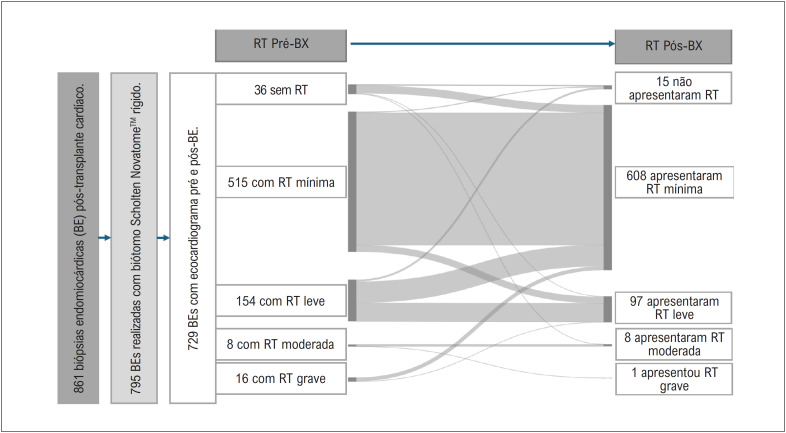
Resultados da regurgitação tricúspide avaliada por eco após 729 biópsias endomiocárdicas em pacientes com transplante cardíaco por meio de abordagem jugular. BE: biópsia endomiocárdica; RT: regurgitação tricúspide.

Houve piora suficiente para se tornar RT moderada ou grave pós-biópsia em apenas 2 (0,27%) procedimentos, e houve uma ligeira alteração de RT mínima para leve pós-BE em 25 (3,42%) procedimentos (Tabela 2 e Figura 3). Não houve piora da RT em 96,3% dos procedimentos de BE.

Houve 1 caso de taquicardia supraventricular sustentada revertida, com administração de adenosina IV 6 mg e 2 casos de trombose venosa crônica da veia jugular direita, o que não impediu a realização do procedimento. Em 729 BEs percutâneas realizadas com um biótomo rígido, não houve perfuração miocárdica, tamponamento cardíaco ou pneumotórax. Uma morte ocorreu dentro de 24 horas após um procedimento de BE. No entanto, a causa não foi identificada.

A taxa geral de complicações, incluindo RT moderada a grave, foi de 0,81%. A taxa de mortalidade foi de 0,14%.

Após a análise dos dados deste registro, em dezembro de 2021, a ecocardiografia pós-procedimento não foi mais realizada rotineiramente, pois não há eventos adversos frequentes ou clinicamente significativos e a segurança em relação à RT foi documentada.

## Discussão

A BE ainda é o procedimento padrão-ouro para detectar rejeição celular após transplante cardíaco. Como cada paciente pós-transplante deve passar por uma série de BEs durante o primeiro ano pós-transplante, a segurança do procedimento tem sido uma preocupação constante. Desde 1970, os biótomos melhoraram substancialmente, e dispositivos flexíveis de uso único com mandíbulas menores foram desenvolvidos, os quais estão associados a uma baixa taxa de complicações.^4^ Um grande registro recente, com aproximadamente 1360 BEs realizadas ao longo de 10 anos em um centro belga, relatou uma taxa geral de complicações de 4,1%.^5^ Ao considerar apenas biópsias do ventrículo direito, a taxa de complicações foi de 3,8%. A maioria das BEs neste registro foi realizada em pacientes pós-transplante (n=937 BEs), usando biótomos flexíveis por acesso femoral. Esse registro também relatou um aumento de 2,5 vezes no risco de complicações por acesso jugular do que com outras abordagens. Bermpeis et al. relataram uma taxa de 0,1% de lesão tricúspide, embora os autores não tenham esclarecido a definição de lesão tricúspide.^5^

Outro registro retrospectivo de 546 procedimentos consecutivos de biópsia cardíaca direita, em pacientes com cardiomiopatia inexplicada de início recente, relatou que a taxa de complicação da inserção da bainha e do procedimento de biópsia foi de 2,7% e 3,3%, respectivamente, com uma taxa geral de complicação de 4,3%.^6^

Nosso estudo demonstrou uma taxa geral significativamente menor de complicações (0,54%) em um tamanho populacional semelhante (n=729 BEs) no mesmo período de dez anos, com a totalidade desses procedimentos sendo realizados sob a técnica de biótomo rígido e 100% dos nossos casos por acesso jugular.

Vale ressaltar que todos os nossos procedimentos foram realizados com técnica de micropunção. Assim, ao considerarmos que cada paciente passou por uma média de 14 procedimentos durante o acompanhamento pós-transplante, todos pela mesma abordagem jugular direita, acreditamos que a técnica de micropunção é recomendável e pode ser uma razão importante para a ausência de complicações vasculares.

Outro possível motivo para a baixa taxa de complicações é um grupo reduzido de cardiologistas dedicados (três), responsáveis por todas as biópsias cardíacas no centro. Portanto, cada operador realiza um número maior de procedimentos do que se as biópsias fossem distribuídas entre todos os cardiologistas intervencionistas.

Nossa coorte de 55 pacientes pós-transplante, que passaram por 729 BEs é possivelmente a maior série de técnica de biótomo rígido por acesso jugular disponível na literatura. Nossa taxa de complicações está abaixo do que foi relatado por outros centros.^4-6^

### Limitações do estudo

O presente trabalho foi realizado retrospectivamente e está sujeito a limitações relacionadas a este desenho, como vieses de medição e memória. Além disso, vale ressaltar que se trata de um estudo de centro único, e a validade externa dos resultados pode ser limitada às diferentes rotinas de cada centro de transplante.

## Conclusão

A BE com biótomo rígido provou ser uma técnica segura. Assim, entendemos que este relatório cumpre seu objetivo de demonstrar, em uma grande amostra, que biópsias endomiocárdicas, realizadas com a técnica de biótomo rígido, não implicam em risco clinicamente relevante de RT após 729 procedimentos em pacientes transplantados cardíacos por abordagem jugular.

A taxa geral de complicações foi de 0,54%.
