# Supplementary material for: Biópsia Endomiocárdica por Meio de Técnica de Biótomo Rígido e Risco de Regurgitação Tricúspide após Transplante Cardíaco
Source: Arq Bras Cardiol. 2024 Oct 17;121(10):e20240223. [Article in Portuguese] doi: 10.36660/abc.20240223 (PMC11634204; doi:10.36660/abc.20240223)
Supplement: Translation

# Translation: Endomyocardial Biopsy Using Rigid Bioptome Technique and the Risk of Tricuspid Regurgitation after Heart Transplantation

**DOI:** 10.36660/abc.20240223i

**Authors:** Luís Beck-da-Silva, Leonardo Hennig Bridi, Bruno S. Matte, Felipe Homem Valle

**Affiliations:** 1 Porto Alegre RS Brazil Serviço de Cardiologia - Hospital de Clínicas de Porto Alegre, Porto Alegre, RS – Brazil; 2 Porto Alegre RS Brazil Departamento de Medicina Interna da Universidade Federal do Rio Grande do Sul (UFRGS), Porto Alegre, RS – Brazil

**Keywords:** Endomyocardial Biopsy, Bioptome, Rigid

## Abstract

Endomyocardial biopsy (EB) is the preferred procedure for post-heart transplant rejection diagnosis. The rigid bioptome technique has been used due to its greater simplicity and has been criticized for the potential risk of tricuspid regurgitation (TR). We aimed to review all the EBs performed by this technique in a tertiary center and estimate the rate of complications and/or aggravation of TR.

Cross-sectional, retrospective, anterograde study. Data were collected from 729 EBs performed in 55 post-heart transplant patients with a rigid Scholten Novatome™ bioptome between September 2012 to March 2022. All EBs were performed via the right jugular vein under local anesthesia and through micro-puncture and ultrasound guidance. A total of 729 procedures had an echocardiography performed before and after the procedures. The estimate of TR was categorized as absent, minimal, mild, moderate, and severe. McNemar's chi-square test was used to analyze the degree of pre- and post-EB TR.

There was a worsening enough to become moderate or severe post-biopsy TR in two (0.27%) procedures, and there was a slight change in TR from minimal to mild TR in 25 (3.42%) procedures. In 729 percutaneous EBs performed with a rigid bioptome, there was no myocardial perforation, cardiac tamponade or pneumothorax. One death occurred within 24 hours after the procedure for an unknown reason.

EB using a rigid bioptome is safe and has not been associated with worsening TR in a follow-up of 729 EBs performed after cardiac transplantation.

The overall complication rate, including moderate to severe TR, was 0.81%. The mortality rate was 0.14%.

## Introduction

Endomyocardial biopsy (EB) has been the standard method for detecting rejection in patients undergoing heart transplantation since the 1970s.^1^ The biopsy technique has evolved over the years following the development and evolution of heart transplantation. The rigid technique was first developed in the mid-1970s as a method of EB developed by Caves and associates^2^ at Stanford University for assessment of cardiac allograft rejection. The new Scholten Novatome™ bioptome combines the long-lasting experience since its development in the 70s and the uniqueness of a disposable, single-use device manufactured with biocompatible material. The Scholten-type bioptome has the advantage of being simple, quick, and safe. However, it requires that the tricuspid valve be crossed with the bioptome forceps for each sample collection. To overcome this potential limitation, a flexible technique was developed using a flexible bioptome that passes through a long introducer sheath. This allows the tricuspid valve to be crossed only once so that all samples are collected through the sheath. However, a comparison of both techniques regarding the risk of tricuspid regurgitation (TR) is lacking.

The present study aims to review all the EBs performed by the Scholten-type bioptome technique in a tertiary center and estimate the rate of complications and/or aggravation of TR.

## Methods

This is a cross-sectional, retrospective, anterograde study.

Patients were selected through a query to the hospital's electronic system (AGHUse). The period for searching for biopsies was from September 2012 to March 2022. The filter used was the procedure performed: EB. From the list containing all biopsies performed, the patient's medical record was opened to verify the reason for performing the myocardial biopsies. Only post-heart transplant patients were included. Data were collected from 861 EBs. Out of these 861 procedures, 795 EBs were performed with a rigid Scholten Novatome™ bioptome in 55 post-heart transplant patients. Our post-heart transplant patients follow a protocol to undergo weekly biopsies in the first month, bi-weekly biopsies up to the third month, monthly biopsies for up to six months and every two months for the first year post-transplant. Extra procedures are included in case of rejection on an as-needed basis. The studied data consists of 729 EBs, which were performed with a rigid Scholten Novatome™ bioptome and had echocardiography performed before and after the EBs (Figure 1). The estimate of TR was evaluated by echocardiography and categorized from absent and minimal, mild, moderate, and severe. Experienced cardiologists performed all echocardiographic exams.

**Figure 1:** Flowchart of post-heart transplant endomyocardial biopsies performed from September 2012 to March 2022 included in the analysis and the degree of pre-biopsy tricuspid regurgitation. TR: tricuspid regurgitation.

All 729 EBs were performed via the right jugular vein under local anesthesia, through micro-puncture technique and under vascular ultrasound guidance (Table 1). Under fluoroscopic control, the right ventricular bioptome, a 50-cm-long catheter with an external diameter of 2.3 mm, was advanced into the bottom third of the right atrium, with its cutting jaws facing to the right. The hand-held part of the bioptome is a modified hemostat that opens and closes the jaws by means of a stiff drive wire, which also gives the bioptome its directability.^3^ The catheter is rotated by its handle so that the tip faces the tricuspid valve orifice, then is slowly advanced through the right ventricle; once the tricuspid valve has been gently crossed, the bioptome is rotated further medially. When the right ventricular septum has been contacted, resistance and the sensation of ventricular contraction are felt by the operator. The jaws are closed, and the bioptome is withdrawn steadily (Figure 2). One or few premature ventricular contractions are usual as the device touches the endomyocardial tissue and at the moment of sample collection. The estimate of TR was evaluated by echocardiography and categorized from absent and minimal, mild, moderate, and severe.

**Table 1:** Baseline characteristics

		All
Age (years) (n=55)		50 (40; 59)
Female sex (n = 55)		19 (35%)
Number of procedures per patient (n=55)		13 (8; 15)
Number of fragments per procedure (n=729)		4 (3; 4)
TR pre-procedure (n=729)		
	Absent or minimal	551 (76%)
	Mild	154 (21%)
	Moderate/severe	24 (3%)

TR: tricuspid regurgitation.

**Figure 2:** Positioning of the rigid bioptome for endomyocardial biopsy.

Data are presented as the mean ±standard deviation, median (percentile 25; percentile 75) or n (%).

A McNemar's Chi-Square test was used to compare the pre- and post-biopsy TR. This test considers that we are comparing the pre and post-changes of the same procedures. Since it is a bi-caudal, the actual value of this p reflects any changes regardless of whether it is toward improvement or worsening of TR (Table 2).

**Table 2:** Distribution of 729 endomyocardial biopsies regarding pre- and post-procedure tricuspid regurgitation

		Post-biopsy TR					
		Absent	Minimal	Mild	Moderate	Severe	Total
Pre-biopsy TR	Absent	4	29	2	1	0	36
	Minimal	4	486	25	0	0	515
	Mild	7	79	68	0	0	154
	Moderate	0	0	0	7	1	8
	Severe	0	14	2	0	0	16
	Total	15	608	97	8	1	729

TR: tricuspid regurgitation.

## Results

The median age of patients was 50 years old (Interquartile range 41, 60).

There was a worsening enough to become moderate or severe post-biopsy TR in only two (0.27%) procedures, and there was a slight change in TR from minimal to mild TR after EB in 25 (3.42%) procedures (Table 2 and Figure 3). There was no worsening of TR in 96.3% of the EB procedures.

**Figure 3:** Outcomes of echo-assessed tricuspid regurgitation following 729 endomyocardial biopsies in heart transplant patients through the jugular approach. EB: endomyocardial biopsy; TR: tricuspid regurgitation.

There was 1 case of sustained supraventricular tachycardia reverted with administration of IV adenosine 6 mg and 2 cases of chronic vein thrombosis right jugular vein, which did not prevent the procedure from being performed. In 729 percutaneous EBs performed with a rigid bioptome, there was no myocardial perforation, cardiac tamponade or pneumothorax. One death occurred within 24 hours after an EB procedure. However, the cause was not identified.

The overall complication rate, including moderate to severe TR, was 0.81%. The mortality rate was 0.14%.

After analysis of data from this registry, in December 2021, post-procedure echocardiography was no longer routinely performed as there are no frequent or clinically significant adverse events, and the safety in relation to TR has been documented.

## Discussion

The EB is still the gold-standard procedure to detect cellular rejection after heart transplantation. As each post-transplant patient must undergo a number of EBs during the first year post-transplant, safety has been a constant concern. Since 1970, the bioptomes have improved substantially, and single-use flexible devices with smaller jaws have been developed that are associated with a low complication rate.^4^ A recent large registry, with approximately 1360 EBs performed over 10 years in a Belgian center, has reported an overall complication rate of 4.1%.^5^ When considering only right ventricle biopsies, the complication rate was 3.8%. The majority of EBs in this registry were performed in post-transplant patients (n=937 EBs) and using flexible bioptomes through femoral access. The Belgian registry also has reported a 2.5-fold increase in the risk of complications by jugular access than with other approaches. Bermpeis et al. reported a 0.1% rate of tricuspid injury, although the authors did not clarify the definition of tricuspid injury.^5^

Another retrospective registry from 546 consecutive right heart biopsy procedures in patients with new onset unexplained cardiomyopathy has reported that the complication rate of sheath insertion and biopsy procedures was 2.7% and 3.3%, respectively. With an overall complication rate of 4.3%.^6^

Our study has demonstrated a significantly lower overall complication rate (0.54%) in a similar population size (n=729 EBs) over the same time frame of ten years, with a totality of these procedures being performed under a rigid bioptome technique and 100% of our cases through jugular access.

It is noteworthy that all our procedures were performed with the micro-puncture technique. Considering that each of our patients has undergone a mean of 14 procedures during post-transplant follow-up, all through the same right jugular approach, we believe that the micro-puncture technique is advisable and is possibly an important reason for not having vascular complications.

Another potential reason for the low complication rate is a dedicated small group of cardiologists (three) who perform all the heart biopsies at the center. Therefore, each operator has a higher volume of procedures than if the biopsies were scattered through all invasive cardiologists.

Our cohort of 55 post-transplant patients who underwent 729 EBs is possibly the largest series of rigid bioptome techniques through jugular access available in the literature. Our complication rate is below what has been reported by other centers.^4-6^

### Study limitations

This work was carried out retrospectively and is subject to limitations related to this design, such as measurement and memory biases. It is also worth noting that this is a single-center study, and the external validity of the results may be limited to the different routines of each transplant center.

## Conclusion

EB using a rigid bioptome proved to be safe. We understand that this report accomplishes its goal of demonstrating that a large sample of endomyocardial biopsies performed with a rigid bioptome technique does not imply a clinically relevant risk of TR following 729 procedures in heart transplant patients through a jugular approach.

The overall complication rate was 0.54%.
